# Supplementary material for: Quantum-enhanced nanodiamond rapid test advances early SARS-CoV-2 antigen detection in clinical diagnostics
Source: Nat Commun. 2025 Oct 2;16:8778. doi: 10.1038/s41467-025-63066-1 (PMC12491541; doi:10.1038/s41467-025-63066-1)
Supplement: Supplementary file 1 — Supplementary Information [file 41467_2025_63066_MOESM1_ESM.pdf]

Quantum-enhanced nanodiamond rapid test advances early SARS-CoV-2 antigen detection in clinical diagnostics

Authors: Alyssa Thomas DeCruz[1,2], Benjamin S. Miller[1,3,4*], Da Huang[1], Max McRobbie[1,2], Felix Donaldson[1], Laura E. McCoy[5], Ciara K. O'Sullivan[6,7], Johannes C. Botha[8,9], Eleni Nastouli[5,8,9], Rachel A. McKendry[1,2*]

Supplementary information

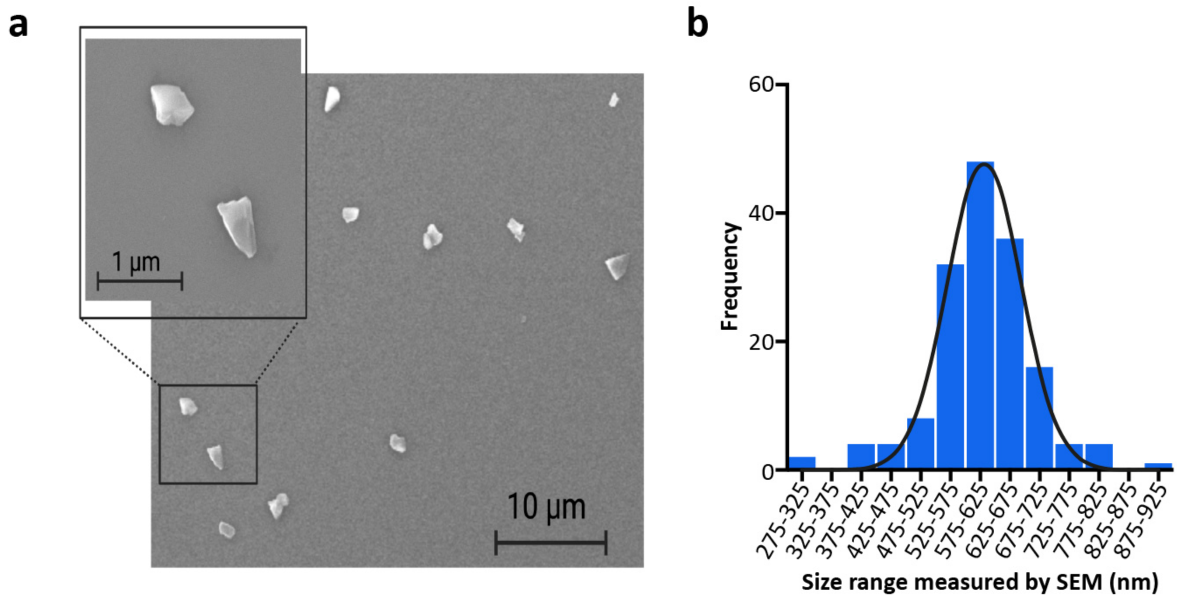

Supplementary Fig. 1: FND particle size characterisation by SEM. (a) Scanning electron microscopy (SEM) image of polyglycerol-coated 600nm FNDs at magnification of 3.21 K X showing monodispersed particles. Call-out box shows particles at magnification 27.03 K X showing cubic morphology of particles. **(b)** Histogram with size dispersion of the 600nm FNDs based on counts (n=156 FND particles) from SEM scans, showing distribution across size ranges from 275nm to 925nm, with peak counts between particles of size 575-625nm. Distribution is fit to a gaussian curve (black line) with mean=606.9 nm and SEM=± 61.7. Source data of particle count are provided as a Source Data file.

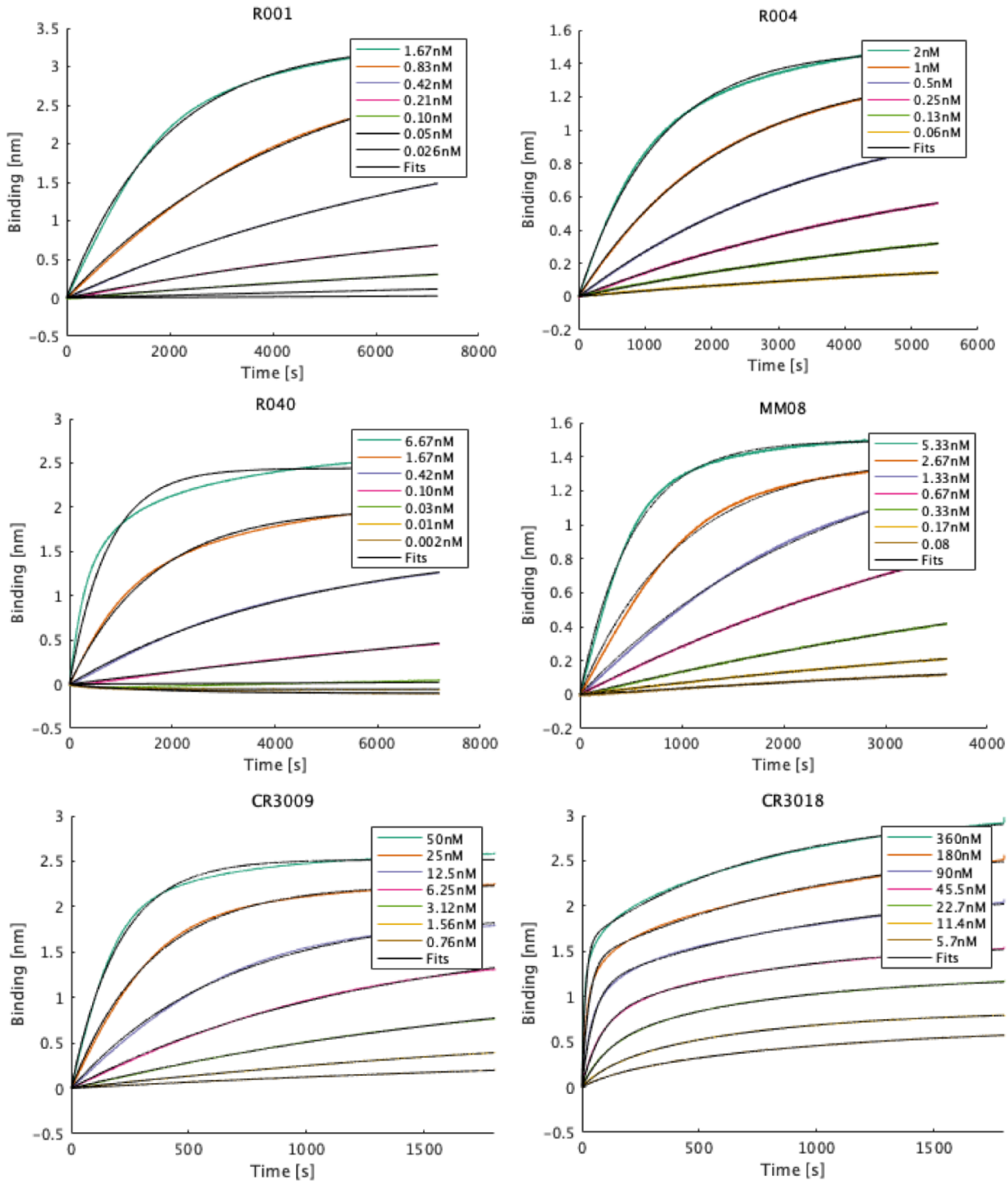

Supplementary Fig. 2: Capture Antibody-Antigen Kinetic Analysis using the ForteBio Octet. Association plot of intensity-time binding for different concentrations of target recombinant nucleocapsid protein for each antibody. Coloured lines represent test data and black lines represent fitted data from Langmuir fit. Antibody CR3018 showed bivalent binding characteristics. Binding curves provided by Sino Biological for antibodies R001 and R040 resulted in K_D values of 0.02 nM and 0.01 nM, respectively[1,2]. Source data are provided as a source data file.

Capture Antibody (Manufacturer, ID)	K_D (nM) [95% CI]	K_{on} (sM) [95% CI]	K_{off} (s^{-1}) [95% CI]
**R001** (Sino Biological[1], RRID: AB_2827974)	0.34 [0.19-0.55]	3.0×10^{5} [2.6-3.5]	2.0×10^{-6} [-33-37]
**R004** (Sino Biological[3], RRID: AB_2827975)	0.22 [0.17-0.27]	3.7×10^{5} [3.5-4.0]	8.5×10^{-5} [6.0-11]
**R040** (Sino Biological[2], RRID: AB_2827976)	0.25 [0.12-0.49]	1.7×10^{5} [-1.79-5.16]	2.3×10^{-4} [-12-16]
**MM08** (Sino Biological[4], RRID: AB_2827978)	0.19 [0.091-0.36]	3.6×10^{5} [3.2-3.9]	1.3×10^{-5} [-6.7-9.4]
**CR3009** (In-house Dr Laura E McCoy, clone ID 03-009[5])	3.3 [2.7-3.9]	9.7×10^{4} [8.8-11]	2.6×10^{-4} [0.71-4.4]
**CR3018** (In-house Dr Laura E McCoy, clone ID 03-018[5])	69 [62-76]	1.6×10^{5} [1.6-1.7]	5.8×10^{-3} [5.0-6.7]

Supplementary Table 1. Summary of capture antibody-antigen kinetic parameters including equilibrium dissociation constant, K_D, association rate, k_{on}, and dissociation rate, k_{off} obtained using BLI. Source data is provided as Source Data file. The MATLAB code for analysis is available in Figshare data repository[6].

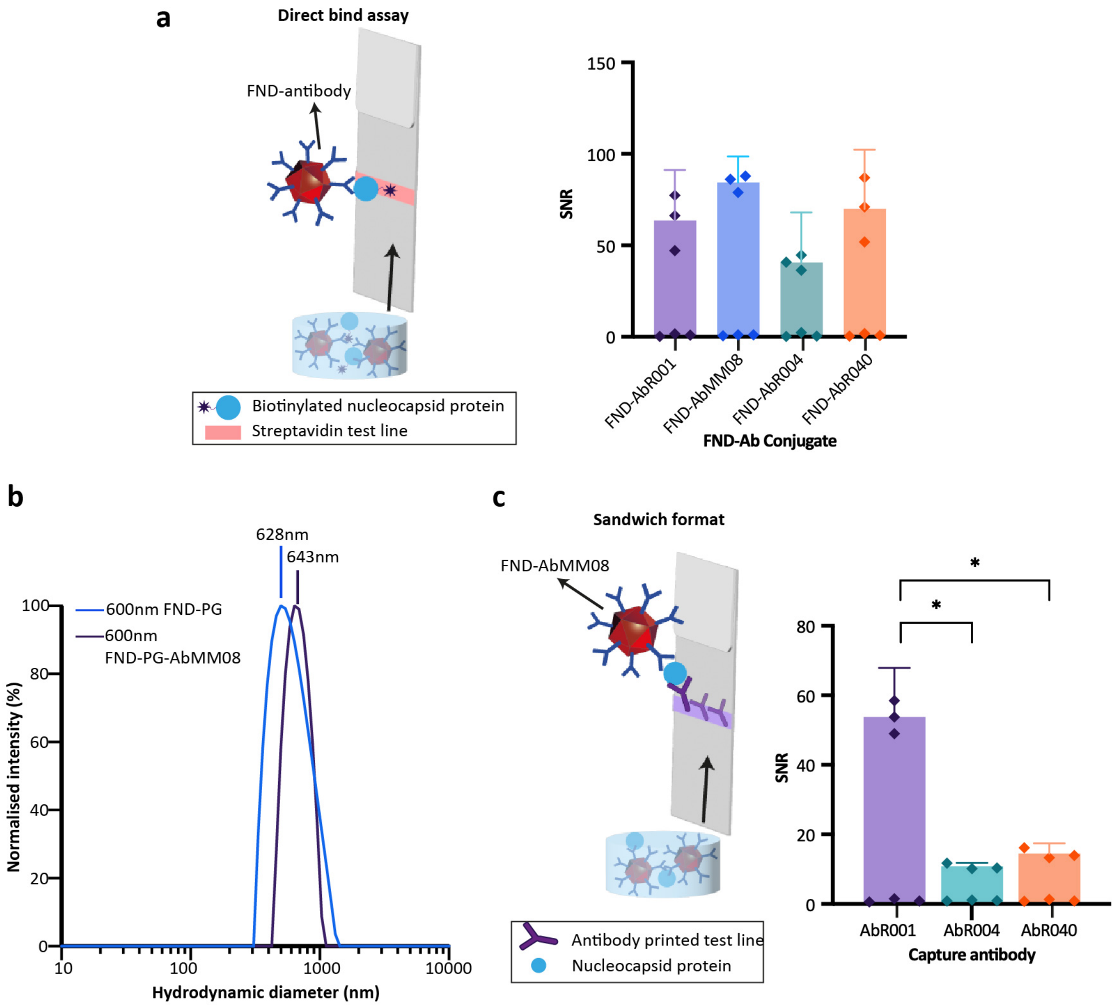

Supplementary Fig. 3: Lateral flow test antibody pair selection. (a) Schematic of direct bind assay and bar plot representing mean signal-to-noise ratio (n=3 test replicates) and SEM using different antibodies functionalised to 600nm FNDs with FND-AbMM08 showing best SNR of 84, but with no statistically significant difference (one-way ANOVA, p-value=0.71, F=0.48, DF=11, post-hoc Tukey's multiple comparisons). Data points represent the SNR of individual test replicates using the mean baseline signal (n=3 test replicates). **(b)** FND size distribution plot using dynamic light scattering showing particle intensity distribution function (%) of both 600nm-FND-PG and antibody functionalised 600nm-FND-PG with hydrodynamic diameters of 628nm (±148), and 643nm (±124). Data presents as intensity trace across 6 process runs and ±SD. **(c)** SNR of different antibody-printed test lines with FND-AbMM08 conjugate. Positive sample was 10ng/mL recombinant protein (n=3 test replicates). Bars represent mean SNR and SEM. Data points represent the SNR of individual test replicates using the mean baseline signal (n=3 test replicates). Statistical significance determined by one-way ANOVA, F=8.2, DF=8, post-hoc Tukey's multiple comparisons, * denotes p-values <0.05: 0.0251 and 0.0363 for AbR001 vs. AbR004 and AbR040, respectively. No significant difference observed between AbR004 and AbR040 (p-value=0.95). Source data is provided as Source Data file.

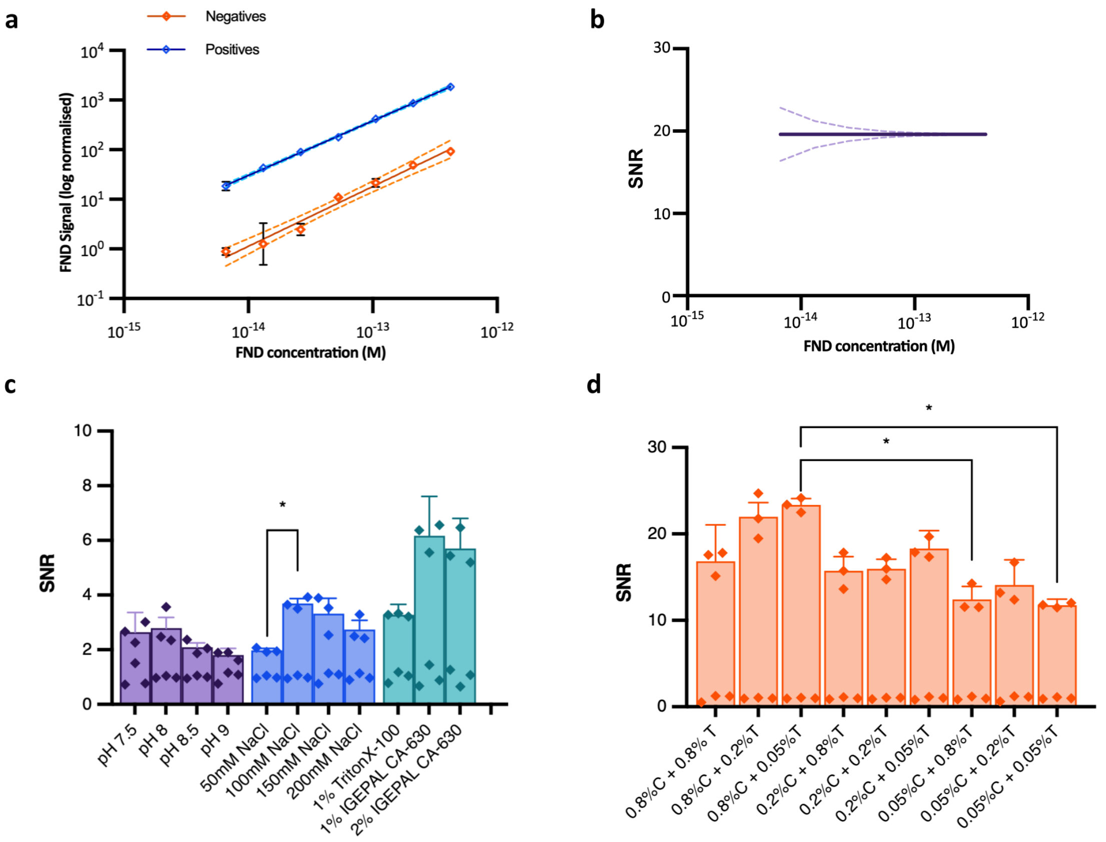

Supplementary Fig. 4: FND-Ag-LFT optimisation (a) FND concentration sweep from 6.6 fM to 42 pM tested on a negative sample (n=3 test replicates) and positive sample (500pg/mL recombinant nucleocapsid protein n=3 test replicates). Data presents as mean and SD (error bars not shown represent SD < 0.05 units). Plots are fitted with linear regressions (solid line) and 95%CI (dashed lines) in Graphpad Prism; negatives (orange) R-squared=0.9545 and positives (blue) R-squared=0.9978. **(b)** SNR found by dividing the fitted linear regressions in (a), resulting in a constant value of ~20. Dashed line represents SD. **(c)** SNR testing different buffer components for viral lysis using whole inactivated virus sample (7.9 x 10^3 $TCID_{50}$/mL) as positive sample (n=3 test replicates) and n=3 negative test replicates. Buffer pH8 was selected and tested with addition of NaCl, where 100 mM NaCl showed a significant difference from 50 mM (one-way ANOVA, p-value=0.0357, F=4.70, DF=8, post-hoc Tukey's multiple comparisons). IGEPAL CA-630 showed better SNRs at both concentrations, although not a statistically significant difference determined by one-way ANOVA, p-value=0.2050, F=2.1, DF=8, post-hoc Tuckey's multiple comparisons. Bars represent mean SNR and SEM. Data points represent the SNR of individual test replicates by mean baseline signal. Buffer was tested independent of additional protein blockers (shown in part d) resulting in overall poorer contrast **(d)** Varying concentrations (%w/v) of Casein (C) and Tween20 (T) in the buffer to determine optimal concentration of blocking components using the SNR of a negative (n=3 test replicates) and positive (500pg/mL recombinant nucleocapsid protein n=3 test replicates). The addition of 0.8% casein and 0.05% Tween20 showed the best SNR of 23, with a significant difference from 0.05%C + 0.8%T (p-value=0.0377) and 0.05%C + 0.05%T (p-value=0.0240) determined by one-way ANOVA, p-value=0.0128, F=3.51, DF=26, Tukey's multiple comparisons. Bars represent mean SNR and SEM. Data points represent the SNR of individual test replicates by mean baseline signal. Source data and full statistical analysis are provided as Source Data file.

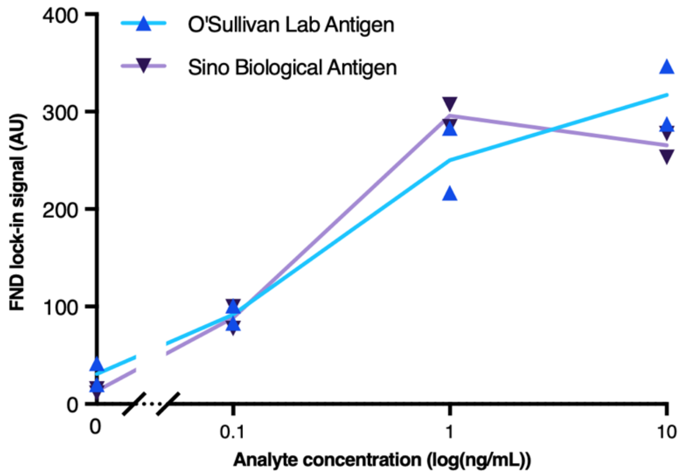

Supplementary Fig. 5: Comparison of two different sources of nucleocapsid protein. Performance with commercially available nucleocapsid protein (Sino Biological Inc.) and nucleocapsid protein developed in-house by the O'Sullivan Lab, tested on the FND SARS-CoV-2 antigen assay showing similar performance across the same antibody pair. Data points represent individual test replicates (n=2) and the solid line tracks the mean. Source data are provided as source data file.

	4PL	Exponential	Langmuir	Stretch exponential
FND Recombinant nucleocapsid protein LoD				
RMSE	0.069	0.057	0.057	0.059
AICc	-54.8	-65.1	-65.0	-62.2
AuNP Recombinant nucleocapsid protein LoD				
RMSE	0.048	0.046	0.046	0.043
AICc	-121.3	-125.7	-125.1	-129.8

Supplementary Table 2: **Statistical comparison of assay LoD fitting models**. A statistical method for determining the limit of detection (LoD) was employed based on methods reported in Holstein et al.,[7] and adapted by Miller et al.[8] Comparison of suitability of LoD fitting models considered[9]: the four-parameter logistic regression, exponential, Langmuir adsorption model, and stretch exponential. A comparison of root mean squared errors (RMSE) and small-sample corrected Akaike Information Criterion (AICc) is shown for all 4 models. All models show excellent RMSE. The 4PL model was the least sensitive. The AICc values show the exponential fit was the best model. Source data are provided as source data file.

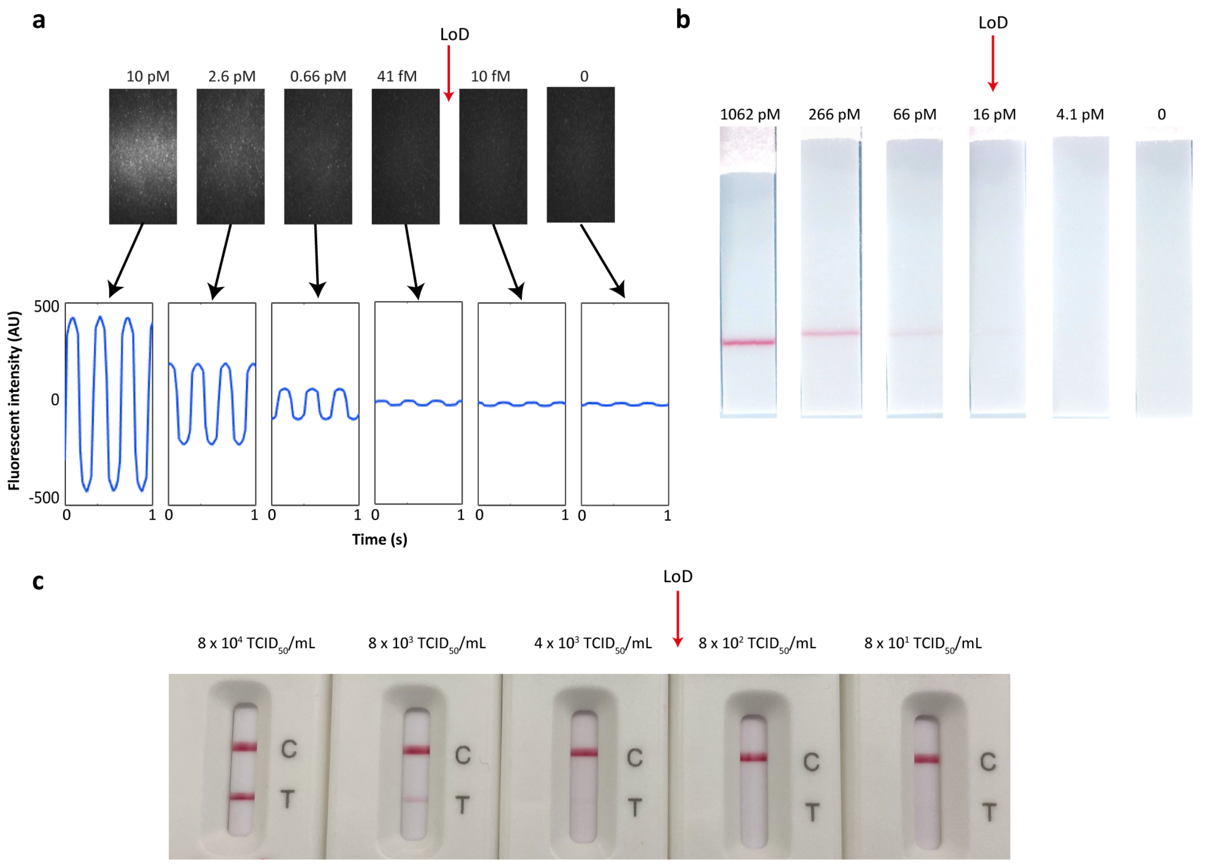

Supplementary Fig. 6: Assay test line analysis across FND LFT, in-house gold LFT and a commercial test. (a) LoD with recombinant nucleocapsid protein on the FND assay (Fig. 2b). Images of FND Ag-LFT test lines from selected concentrations of serial dilution with corresponding fluorescent intensity time series plots (bottom) showing detectable periodic signal after test line is no longer visible in the images. Arrow shows the approximate LoD calculated from the exponential fit (Fig. 2b). Processed test line images are available at Figshare data repository[6]. Modulation time series plot source data are provided as source data file. **(b)** Select concentrations of strips from AuNP assay limit of detection with recombinant nucleocapsid protein (single test strip shown from n=3 test replicates). AuNP assay strips were ran for 15mins and imaged by camera in a light-controlled box. Images of the test line were then analysed through MATLAB using a pixel-wise line intensity plot. Arrow points to the calculated LoD from exponential fitting curve. Unprocessed images of all test strips have been deposited in the Figshare data repository[6] **(c)** Images of FlowFlex SARS-CoV-2 Ag assay tested with selected concentrations of gamma irradiated wild-type viral isolate following the product insert protocol. Arrow indicates approximate concentration of lowest detectable test line at ~10^3 $TCID_{50}$/mL.

Reference	Sensor type	LoD	Clinical evaluation high viral load (Ct≤25 or 10^6 copies/mL) sensitivity
Chen et al. (2022)[10]	Multiplex fluorescent quantum dot LFT	10 pg/mL	No clinical samples tested
Grant et al. (2021)[11] Bachman et al. 2021)[12]	Latex bead LFT	200 $TCID_{50}$/mL	92% (n=72)
Wang et al. (2021)[13]	Magnetic quantum dot LFT	1 pg/mL (direct mode)	No clinical samples tested
Wei-Wen Hsiao et al. (2022)[14]	Fluorescent nanodiamond LFT (magnetic modulation)	1.94 ng/mL	No clinical samples tested
Gupta et al. (2023)[15]	Fluorescent gold nanorod LFT	212 pg/mL	97.5% (n=40)
This work	Fluorescent nanodiamond LFT (MW modulation)	0.78 pg/mL	100% (n=30)

Supplementary Table 3: **Comparison of assay sensitivities reported in literature**. Summary of SARS-CoV-2 Ag-LFTs targeting nucleocapsid protein reported in literature with their assay limit of detection.

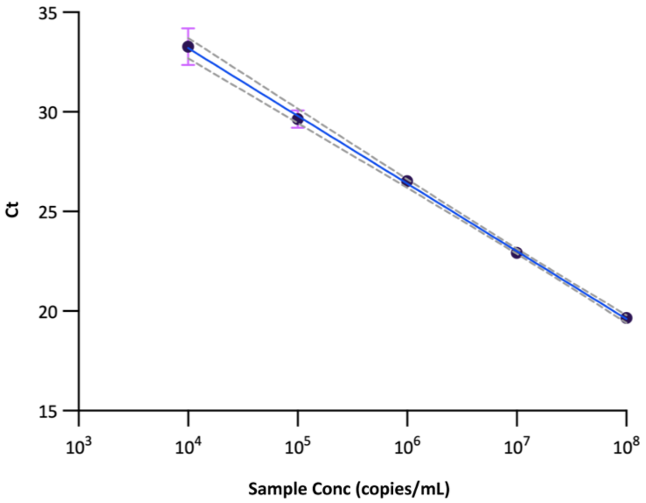

Supplementary Fig. 7: RT-qPCR standard curve. RT-qPCR standard curve of standard Omicron viral RNA (n=3 test replicates) to convert Ct to copies/mL. The data were fitted to a Bayesian linear regression (solid blue line with grey dashed lines for 95% credible interval) weighted by the uncertainty in Ct. The fit, along with error propagation was used to calculate copies/mL and associated errors from Ct values from clinical samples. Errors were calculated by combining the uncertainty in the model (sampling from the posterior to get the distribution of copies/mL values for a given Ct value), and the uncertainty in the measured Ct value (from interpolating the relationship between Ct and Ct standard error). Circles with error bars represent mean and standard deviation. Source data are provided as source data file.

Area under the ROC curve	All samples	Samples Ct ≤ 30	Samples Ct ≤ 25
Area	0.92	0.98	1.00
Std. Error	0.03	0.02	0.00
95% confidence interval	0.86 to 0.99	0.94 to 1.00	1.000 to 1.000
P value	8.98×10^{-12}	4.84×10^{-13}	2.60×10^{-12}
**Data**			
Controls (Negative)	37	37	37
Patients (Signal)	53	41	30

Supplementary Table 4: Results summary of clinical sensitivity and specificity ROC analysis. ROC curve (95% CI calculation with Wilson/Brown method) analysed for all samples (n=53 distinct clinical samples) and sub-categories for Ct ≤ 30 and Ct ≤ 25. Resulting p-values show statistically significant area under the curve (AUC) from an AUC =0.5 (no discriminatory power). Source data are provided as source data file.

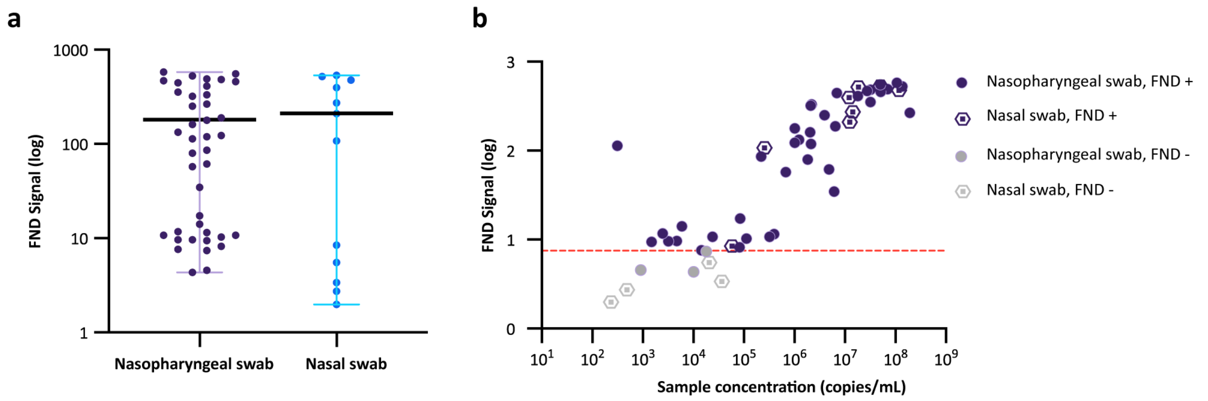

Supplementary Fig. 8: Clinical sample matrix stratification by nasal and nasopharyngeal swab type. (a) Box plot of positive clinical sample FND results stratified by nasopharyngeal swab (n=41 distinct clinical samples) and nasal swab (n=12 distinct clinical samples) type showing mean FND signal (black line) and range. No significant difference between mean FND signal was observed (unpaired two tailed t-test p-value=0.6457, T=0.4625, DF=51). **(b)** Visual representation of FND results by clinical sample viral load (copies/mL) and swab type showing distribution of viral loads in nasal swabs (hexagon, n=12 distinct clinical samples) and nasopharyngeal swabs (circles, n=41 distinct clinical samples). No significant difference between mean viral load across sample type determined by unpaired two-tailed t-test, p-value=0.9886, T=0.01432, DF=51). Red dashed line represents FND negative signal threshold. Grey data points represent FND assay negative results and purple represents FND assay positive results. Source data are provided as source data file.

	High viral load ($\geq 10^6$ copies/mL)	moderate viral load (10^5-10^4 copies/mL)	Full range (10^2-10^8 copies/mL)
FND sensitivity	100%	90%	87%
AuNP sensitivity	93%	20%	57%

Supplementary Table 5: **Estimating AuNP Ag-LFT clinical sensitivity compared to FND Ag-LFT sensitivity evaluation.** Summary of assay sensitivity grouped by viral load of clinical samples using extrapolated threshold calculated for the AuNP assay. High viral load samples n=29, moderate viral load samples n=10, full range n=53. Sensitivities were calculated non-cumulatively, as opposed to Fig. 3a grouped by Ct with cumulative sensitivities. The sensitivities calculated within each viral load category were used for modelling analysis accompanying Fig.4c.

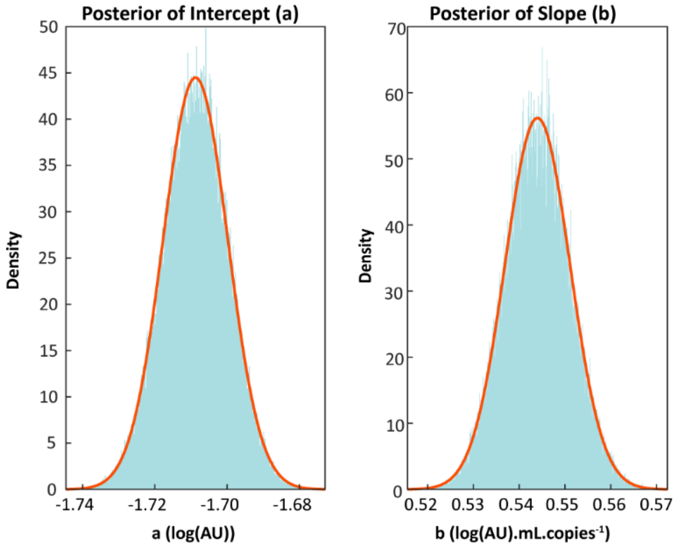

Supplementary Fig. 9: Clinical sample Bayesian regression posterior distributions. Posterior distributions for the intercept and slope for the symmetrical Bayesian linear regression with errors-in-variables in x and y shown in Fig. 3b. See methods for priors and likelihood functions. Blue histograms show Markov chain Monte Carlo sampling of the posteriors and the red lines show Gaussian distributions with means and standard deviations calculated from the distributions.

Estimating number of patients detected in a single day at peak pandemic

To put the spin-enhanced LFT diagnostic advantage in context of patient numbers at the population level, we applied the respective assay sensitivity to the total number of confirmed COVID-19 cases in a single day at peak pandemic to estimate how many more patients the FND assay may have detected than the AuNP assay. In this case, the daily reported case number does not include stratification of individual patient data by day of symptom onset or viral load, therefore we applied the total assay sensitivity for this calculation. The sensitivity was 87% and 57% for the FND assay and AuNP assay, respectively (Supplementary Information Table 5). This analysis assumes that the sensitivity calculated from our clinical sample data set is generally representative of the patient population.

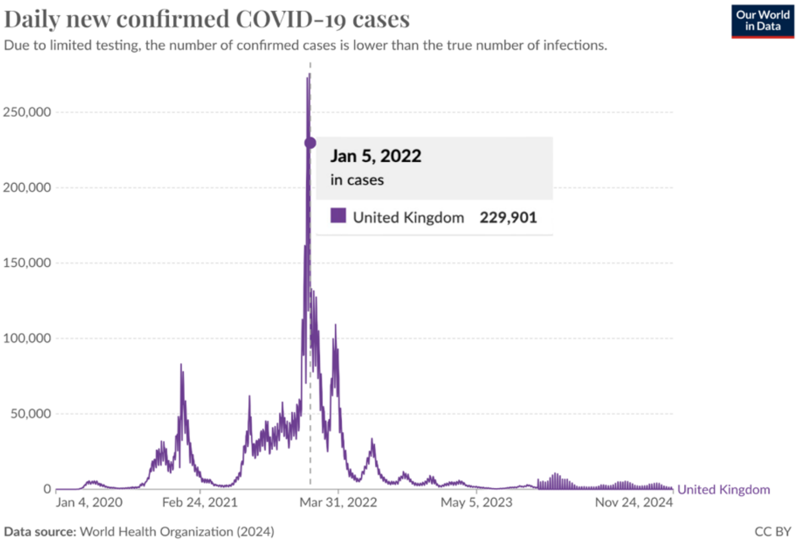

Supplementary Fig. 10: World Health Organisation data tracking daily new confirmed COVID-19 cases in the United Kingdom over the course of the pandemic (Jan 2020 to Nov 2024). Marker indicated on Jan 5th corresponds to the Omicron peak reaching nearly 230,000 new cases in a single day, in which approximately 69,400 more patients may have been detected by the FND assay than the AuNP assay. This assumes our clinical sample set is representative of the population viral load distribution at the peak of the Omicron wave. Graph downloaded from https://ourworldindata.org/covid-cases.

Assessing performance retention transitioning to an integrated point-of-care platform

The assay presented has been designed for greater flexibility in design and optimisation throughout the initial stages of development in a lab-based setting. This section addresses the feasibility of retaining similar performance in a fully integrated lateral flow platform. These considerations include (i) adequate reaction kinetics for on-pad reagent delivery in cassette format (ii) immediate read-out of strip following 15min run time (without drying) for fast time-to-results and (iii) feasibility of cost-effective, portable fluorescence read-out device.

a) Reaction kinetics study: Demonstrating equivalent assay performance with direct strip application.

This study shows feasibility of eliminating the 10-minute incubation of reagents prior to adding the lateral flow strip, which more closely represents the binding interactions that occur in a cassette-based lateral flow format as complexes are formed and captured immediately during flow up the membrane. Strips were tested with a wait time from 0-10 mins at 1 min intervals, evaluating the impact on the SNR from a low positive (31 pg/mL, n=3 test replicates) and negative sample (n=3 test replicates) over time. A low analyte concentration (31 pg/mL) was chosen for this study to reflect a case where complex formation/binding is less favourable and therefore more likely to observe potential impact on performance than at high concentrations where binding time has diminished impact.

The assay was performed as follows: 5μL of the FNDs (26 fM) + 49μL of sample diluted in running buffer + 1μL of bAb01 (513nM) was added to wells in a 96-well plate and allowed to bind for 0-10 mins. Strip were added to the wells and run for 15 mins, followed by test line read-out of dry strips. Time 0 mins corresponds to application of the lateral flow strip immediately after adding reagents to the wells and 10 min wait time is representative of the protocol used throughout this work. No significant difference in SNR was observed across the time intervals tested (Supplementary Fig. 11), indicating the complex formation in solution does not impact effective capture at the test line. In future, eliminating the “binding time” step for this assay can reduce assay time to the standard ~15 min lateral flow test run time without impacting performance.

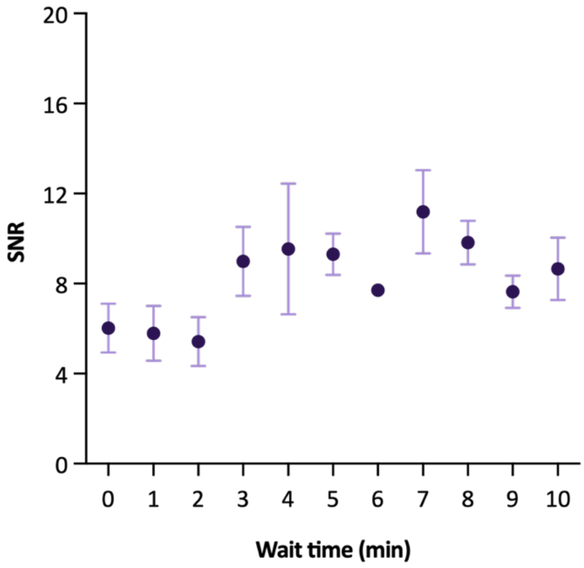

Supplementary Fig. 11: Evaluating impact of reagent incubation step on assay performance (a) The SNR of negative (n=3 test replicates) and low positive (31 pg/mL, n=3 test replicates) tested at 1 min time intervals where at time 0, the lateral flow strips were ran immediately after addition of capture/detection reagents. Data points represent mean SNR and SEM, showing no significant difference between mean SNRs across the time interval (one-way ANOVA, p-value=0.1479, F= 1.68, DF=32, post-hoc Tukey's multiple comparisons) and a gradient not significantly different from zero using a linear regression fit (95%CI: -0.0089 to 0.67). Source data are provided as source data file.

b) Impact of lateral flow test strip drying on FND lock-in signal

This short study looked at the effect of reading wet lateral flow strips on the resonator-based read-out platform. This would allow for read-out of lateral flow strips immediately following running, reducing the total assay time to fit the point-of-care criteria. The key potential confound for wet strips is the shift in the resonator's resonant frequency (water has a high dielectric constant).

Test strips from a positive (n=2 test replicates) and negative (n=3 test replicates) sample were measured using lock-in analysis at 1-minute time intervals from 0-60 mins from the end of strip running. We observed a ~5 point reduction in lock-in values in the first ~10 minutes of read-out, likely due to FNDs still washing up the strip. This happens in all LFTs, not just nanodiamond LFTs. This is a higher proportion of the final lock-in value for the negatives, as the signal is lower, so there is a net effect on SNR: lower immediately after reading. It will have a minimal effect on sensitivity and can be mitigated with a differential readout (normalising to strip background).

Over the subsequent period of about 5-minutes, the signals rise in both positive and negatives tests as the strips dry. The overall effect on SNR is therefore zero. This can therefore be mitigated by taking ratiometric measurements of test line and background (analogous to test line and signal), or test line and control line. Finally, the resonator could be tuned to account for the changes in resonant frequency with wet strips (either in real-time or preset).

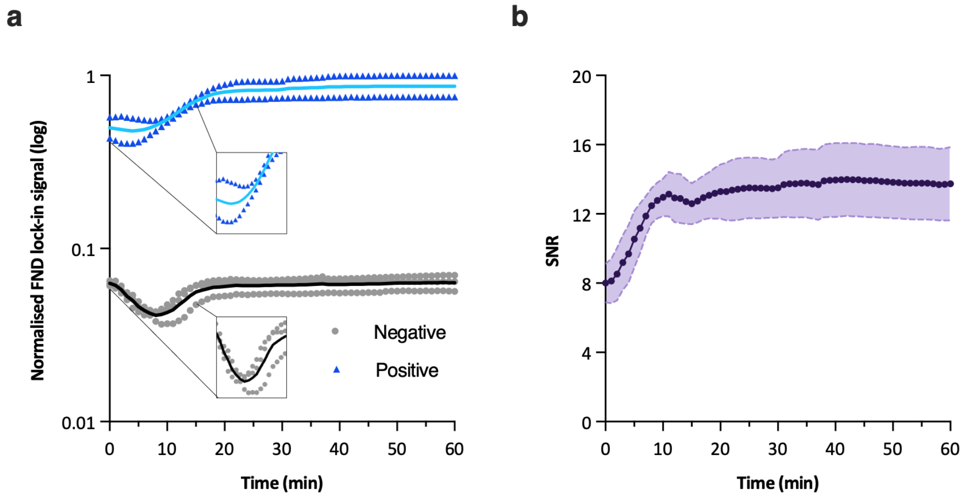

Supplementary Fig. 12: Evaluating the impact of wetting on lateral flow lock-in analysis (a) The normalised FND lock-in signal of negatives (grey, n=3 test replicates) and positives (blue, n=2 test replicates), measured at 1-minute intervals over 60 minutes. Data points represent individual test replicates and solid line represents the mean. The effect of wetting reduced lock-in signal of positive samples by ~40% of its maximum value at time 0, with a coefficient of variation (CV) = 18.3% across all time points. Minimal effect on the negatives was observed CV=11.2% across all time points. Inset shows region from 0-15 minutes on a linear scale, where an initial decrease in lock-in signal is observed, likely due to FNDs washing away while the strip is still wet and solution is still moving across the strip. This is then counterbalanced by the strip drying, increasing signal again. In the positive sample, many of these particles bind to the test line rather than flowing past, giving a smaller dip. **(b)** The signal-to-noise ratio was calculated from the means in (a), with a minimum SNR of 8 (± 1.1) at time 0 and reaching a plateau ~11 mins with SNR=13 (± 1.2). The SNR has variation of 11.2% across 60 mins. Data presents as mean ± SEM. Source data are provided as source data file.

c) Estimated costs of a proposed portable fluorescent reader

Although the measurements in this paper were performed using a fluorescence microscope, they could in principle be performed with a low-cost device. The feasibility of a portable fluorescent reader with modulation capabilities is a key component to the translation of the FND platform to a point-of-care setting. A portable, smartphone-based read-out system meets point-of-care criteria using cost effective 3D-printed housing, miniaturised electronics, and small resonator components allow for integration into a portable device that can be deployed in the field and at the point-of-care including doctors' offices and pharmacies. A costing summary of a proposed prototype is shown in Supplementary Information Table 6:

Device	Description	Cost (£)
FND prototype smartphone reader	Resonator	6
	30mW laser	93
	Lens & filters	221
	Cables & 3D printed material	25
	Voltage controlled oscillator & chip	65
	amplifier	137
	Power bank	20
	Smartphone (LG-G7)	345
	**Total**	**912**

Supplementary Information Table 6: Estimated costing of a proposed FND portable reader with smartphone connectivity in development.

Supplementary References

1. Sino Biological. *Datasheet Catalog Number 40143-R001*. https://www.sinobiological.com/antibodies/cov-nucleocapsid-40143-r040 (2020).
2. Sino Biological. *Datasheet Catalog Number 40143-R040*. https://www.sinobiological.com/antibodies/cov-nucleocapsid-40143-r040 (2020).
3. Sino Biological. *Datasheet Catalog Number 40143-R004*. https://www.sinobiological.com/antibodies/cov-nucleocapsid-40143-r004 (2020).
4. Sino Biological. *Datasheet Catalog Number 40143-MM08*. https://www.sinobiological.com/antibodies/cov-nucleocapsid-40143-mm08 (2021).
5. van den Brink, E. N. *et al.* Molecular and biological characterization of human monoclonal antibodies binding to the spike and nucleocapsid proteins of severe acute respiratory syndrome coronavirus. *J Virol* **79**, 1635–1644 (2005).
6. Thomas DeCruz, A., Miller, B. S. & McKendry, R. A. Quantum-enhanced nanodiamond rapid test advances early SARS-CoV-2 antigen detection in clinical diagnostics- Data sets. *Figshare* Preprint at (2025).
7. Holstein, C. A., Griffin, M., Hong, J. & Sampson, P. D. Statistical Method for Determining and Comparing Limits of Detection of Bioassays. *Anal Chem* **87**, 9795–9801 (2015).
8. Miller, B. S. *et al.* Spin-enhanced nanodiamond biosensing for ultrasensitive diagnostics. *Nature* **587**, 588–593 (2020).
9. Miller, B. S. *et al.* Sub-picomolar lateral flow antigen detection with two-wavelength imaging of composite nanoparticles. *Biosens Bioelectron* **207**, 956–5663 (2022).
10. Chen, W. *et al.* An integrated fluorescent lateral flow assay for multiplex point-of-care detection of four respiratory viruses. *Anal Biochem* **659**, 114948 (2022).
11. Grant, B. D. *et al.* A SARS-CoV-2 coronavirus nucleocapsid protein antigen-detecting lateral flow assay. *PLoS One* **16**, e0258819 (2021).
12. Bachman, C. M. *et al.* Clinical validation of an open-access SARS-COV-2 antigen detection lateral flow assay, compared to commercially available assays. *PLoS One* **16**, e0256352 (2021).
13. Wang, C. *et al.* Ultrasensitive and Simultaneous Detection of Two Specific SARS-CoV-2 Antigens in Human Specimens Using Direct/Enrichment Dual-Mode Fluorescence Lateral Flow Immunoassay. *ACS Appl Mater Interfaces* **13**, 40342–40353 (2021).
14. Wei-Wen Hsiao, W. *et al.* Fluorescent nanodiamond-based spin-enhanced lateral flow immunoassay for detection of SARS-CoV-2 nucleocapsid protein and spike protein from different variants. *Anal Chim Acta* **1230**, 340389 (2022).
15. Gupta, R. *et al.* Ultrasensitive lateral-flow assays via plasmonically active antibody-conjugated fluorescent nanoparticles. *Nat Biomed Eng* **7**, 1556–1570 (2023).
