# Supplementary material for: Quantum-enhanced nanodiamond rapid test advances early SARS-CoV-2 antigen detection in clinical diagnostics
Source: Nat Commun. 2025 Oct 2;16:8778. doi: 10.1038/s41467-025-63066-1 (PMC12491541; doi:10.1038/s41467-025-63066-1)
Supplement: Supplementary file 2 — Reporting Summary [file 41467_2025_63066_MOESM2_ESM.pdf]

nature portfolio

Corresponding author(s): Rachel A McKendry, Benjamin S. Miller

Last updated by author(s): Aug 5, 2025

Reporting Summary

Nature Portfolio wishes to improve the reproducibility of the work that we publish. This form provides structure for consistency and transparency in reporting. For further information on Nature Portfolio policies, see our Editorial Policies and the Editorial Policy Checklist.

Statistics

For all statistical analyses, confirm that the following items are present in the figure legend, table legend, main text, or Methods section.

n/a	Confirmed	
☐	☒	The exact sample size (*n*) for each experimental group/condition, given as a discrete number and unit of measurement
☐	☒	A statement on whether measurements were taken from distinct samples or whether the same sample was measured repeatedly
☐	☒	The statistical test(s) used AND whether they are one- or two-sided<br>*Only common tests should be described solely by name; describe more complex techniques in the Methods section.*
☒	☐	A description of all covariates tested
☐	☒	A description of any assumptions or corrections, such as tests of normality and adjustment for multiple comparisons
☐	☒	A full description of the statistical parameters including central tendency (e.g. means) or other basic estimates (e.g. regression coefficient) AND variation (e.g. standard deviation) or associated estimates of uncertainty (e.g. confidence intervals)
☐	☒	For null hypothesis testing, the test statistic (e.g. F, t, r) with confidence intervals, effect sizes, degrees of freedom and P value noted<br>*Give P values as exact values whenever suitable.*
☐	☒	For Bayesian analysis, information on the choice of priors and Markov chain Monte Carlo settings
☒	☐	For hierarchical and complex designs, identification of the appropriate level for tests and full reporting of outcomes
☒	☐	Estimates of effect sizes (e.g. Cohen's *d*, Pearson's *r*), indicating how they were calculated

Our web collection on statistics for biologists contains articles on many of the points above.

Software and code

Policy information about availability of computer code

Data collection	Antibody BLI data collection on Octet RED96 Data Acquisition software 8.2, test line image data collection HCImage live software v4.4.1.0, RT-qPCR data collection on QuantStudio v1.5.1, DLS data on Anton Paar Litesizer 500 Kalliope version 2.22.1
Data analysis	The MATLAB 2023a software for fitting robust detection limits (LODs) v1.4 and confidence intervals to serial dilution data is available at https://github.com/bensmiller/detection-limit-fitting/. GraphPad Prism 7 and MATLAB 2023a was used for plotting and statistical analysis. ImageJ 1.53u used for SEM particle size analysis. qPCR analysis Design & Analysis 2.6.0

For manuscripts utilizing custom algorithms or software that are central to the research but not yet described in published literature, software must be made available to editors and reviewers. We strongly encourage code deposition in a community repository (e.g. GitHub). See the Nature Portfolio guidelines for submitting code & software for further information.

Data

Policy information about availability of data

All manuscripts must include a data availability statement. This statement should provide the following information, where applicable:
- Accession codes, unique identifiers, or web links for publicly available datasets
- A description of any restrictions on data availability
- For clinical datasets or third party data, please ensure that the statement adheres to our policy

The Source data are provided with this paper. Larger datasets are available in Figshare with identifier: https://doi.org/10.6084/m9.figshare.29490068. These are: the mean pixel data of each 750 image data set for each sample analysed for limit of detection analysis (Fig. 2b,d; Supplementary Fig. 6) and all clinical samples

tested (Fig. 3), and raw data for antibody BLI kinetics plot (Fig. 2a, Supplementary Fig.2, Supplementary Table 1). We have deposited the mean pixel data (simply the mean of each image, which is used for all analysis) rather than raw images (raw images are never used directly) for all samples tested in this work due to the large file size (750 images per replicate). The raw image data can be directly provided upon request of the corresponding author(s). We will process all requests within two weeks.

Thomas DeCruz, A., Miller, B. S. & McKendry, R. A. Quantum-enhanced nanodiamond rapid test advances early SARS-CoV-2 antigen detection in clinical diagnostics-Data sets. Figshare. (2025). doi:/10.6084/m9.figshare.29490068

Research involving human participants, their data, or biological material

Policy information about studies with human participants or human data. See also policy information about sex, gender (identity/presentation), and sexual orientation and race, ethnicity and racism.

Reporting on sex and gender	Sex and gender information were not disclosed to or considered by investigators in this study. The accuracy of the test was assessed based solely on the biological characteristics of the biospecimens (swab sample) and their corresponding laboratory results, therefore sex and gender variables were not relevant to the study objectives
Reporting on race, ethnicity, or other socially relevant groupings	Race and ethnicity information were not disclosed to or considered by investigators in this study. The accuracy of the test was assessed based solely on the biological characteristics of the biospecimens (swab sample) and their corresponding laboratory results, which are not influenced by race or ethnicity. Therefore these variables were not relevant to the study objectives
Population characteristics	Population characteristics other than those relevant to the diagnostic test were not disclosed to or considered by investigators in this study. The focus of the study was to evaluate the diagnostic accuracy of the test based on the biological properties of the sample, independent of other patient characteristics
Recruitment	Clinical samples were collected from various individuals at UCLH (including travelers, healthcare workers, and patients) following standard operating procedure. The recruitment process did not involve direct participant interaction with this study, therefore potential biases related to self-selection or other factors are not applicable
Ethics oversight	University College London Hospital governance committee IRB no. NDU-VIR_131/13122022); University College London Infection DNA Bank 2022 (IRAS ID 320050).

Note that full information on the approval of the study protocol must also be provided in the manuscript.

Field-specific reporting

Please select the one below that is the best fit for your research. If you are not sure, read the appropriate sections before making your selection.

☒ Life sciences ☐ Behavioural & social sciences ☐ Ecological, evolutionary & environmental sciences

For a reference copy of the document with all sections, see nature.com/documents/nr-reporting-summary-flat.pdf

Life sciences study design

All studies must disclose on these points even when the disclosure is negative.

Sample size	No calculation was performed to determine sample size. A sample size of n=3 distinct samples at 7 (or more) test concentrations was selected based on previous studies using similar methodologies typically utilising a sample size of 3-5 replicates over a 5-point standard curve, demonstrating this range of replicates provides sufficient data for accurate curve fitting and subsequent analysis.
Data exclusions	No data was excluded in the analyses of this study
Replication	Key findings on characterisation and analytical limit of detection were replicated in independent experiments three times following reported methods. Replication studies were successful. Clinical evaluation with residual samples could not be replicated due to insufficient sample volume.
Randomization	Residual clinical samples were randomly selected by individuals at UCLH to include a random selection of positive and negative samples, broadly representative of the normal population
Blinding	Blinded residual clinical samples were assigned a unique identification number by individuals at UCLH for transfer to investigators for initial data collection and analysis. Post-analysis, investigators received unblinded sample identification number with corresponding clinical results

Reporting for specific materials, systems and methods

We require information from authors about some types of materials, experimental systems and methods used in many studies. Here, indicate whether each material, system or method listed is relevant to your study. If you are not sure if a list item applies to your research, read the appropriate section before selecting a response.

Materials & experimental systems

n/a	Involved in the study
☐	☒ Antibodies
☒	☐ Eukaryotic cell lines
☒	☐ Palaeontology and archaeology
☒	☐ Animals and other organisms
☒	☐ Clinical data
☒	☐ Dual use research of concern
☒	☐ Plants

Methods

n/a	Involved in the study
☒	☐ ChIP-seq
☒	☐ Flow cytometry
☒	☐ MRI-based neuroimaging

Antibodies

Antibodies used

Sino Biological: Cat#40143-R001-B, clone ID 001,Lot#HP15JA2004; Cat#40143-MM08, clone ID 08, Lot# MA14DE0202, RRID AB_2827978; Cat#40143-R001, clone ID 001, RRID AB_2827974; Cat#40143-R004, clone ID 004, RRID AB_2827975; Cat#40143-R040, clone ID 040, RRID AB_2827976. Clone ID CR3009; Clone ID CR3018. Test concentrations ranging from 1 mg/mL down to 0.5pg/mL

Validation

The antibodies were validated by the manufacturer (Sino biological) on WB, ELISA,IHC-P, and FCM, validation data is publicly available through the manufacturers datasheet and certificate of analysis (https://www.sinobiological.com/antibodies). Antibodies CR3009 and CR3018 original source is referenced in Supplementary Table 1 with reference: van den Brink, E. N. et al. Molecular and biological characterization of human monoclonal antibodies binding to the spike and nucleocapsid proteins of severe acute respiratory syndrome coronavirus. J Virol 79, 1635–1644 (2005).

Plants

Seed stocks

Report on the source of all seed stocks or other plant material used. If applicable, state the seed stock centre and catalogue number. If plant specimens were collected from the field, describe the collection location, date and sampling procedures.

Novel plant genotypes

Describe the methods by which all novel plant genotypes were produced. This includes those generated by transgenic approaches, gene editing, chemical/radiation-based mutagenesis and hybridization. For transgenic lines, describe the transformation method, the number of independent lines analyzed and the generation upon which experiments were performed. For gene-edited lines, describe the editor used, the endogenous sequence targeted for editing, the targeting guide RNA sequence (if applicable) and how the editor was applied.

Authentication

Describe any authentication procedures for each seed stock used or novel genotype generated. Describe any experiments used to assess the effect of a mutation and, where applicable, how potential secondary effects (e.g. second site T-DNA insertions, mosiacism, off-target gene editing) were examined.
